# Real-world evidence from a retrospective study on suicide during depression: clinical characteristics, treatment patterns and disease burden

**DOI:** 10.1186/s12888-024-05726-y

**Published:** 2024-04-19

**Authors:** Han Wang, Nan Lyu, Juan Huang, Bingbing Fu, Lili Shang, Fan Yang, Qian Zhao, Gang Wang

**Affiliations:** 1 Beijing Key Laboratory of Mental Disorders, Beijing Anding Hospital, National Clinical Research Center for Mental Disorders & National Center for Mental Disorders, Capital Medical University, 5 Ankang Hutong Road, Xicheng District, 100088 Beijing, China; 2 The Advanced Innovation Center for Human Brain Protection, Capital Medical University, 100069 Beijing, China

**Keywords:** Major depressive disorder, Suicide, Real-world study, Treatment pattens

## Abstract

**Background:**

Suicide stands as both a primary symptom and the direst outcome of major depressive disorder (MDD). The scarcity of effective treatment strategies makes managing MDD patients with suicide especially challenging. Hence, it is crucial to investigate disease characteristics and efficacious therapeutic strategies for these patients, drawing insights from disease databases and real-world data.

**Methods:**

In this retrospective study, MDD patients hospitalized between January 2013 and December 2020 were investigated using Electronic Health Records (EHR) data from Beijing Anding Hospital. The study enrolled 4138 MDD patients with suicidal ideation or behavior (MDS) and 3848 without (MDNS). Demographic data, clinical attributes, treatment approaches, disease burden, and re-hospitalization within one year of discharge were extracted and compared.

**Results:**

Patients in the MDS group were predominantly younger and female, exhibiting a higher prevalence of alcohol consumption, experiencing frequent life stress events, and having an earlier onset age. Re-hospitalizations within six months post-discharge in the MDS group were significantly higher than in the MDNS group (11.36% vs. 8.91%, *p* < 0.001). Moreover, a more considerable fraction of MDS patients underwent combined electroconvulsive therapy treatment (56.72% vs. 43.71%, *p* < 0.001). Approximately 38% of patients in both groups were prescribed two or more therapeutic regimes, and over 90% used antidepressants, either alone or combined. Selective serotonin reuptake inhibitors (SSRIs) were the predominant choice in both groups. Furthermore, antidepressants were often prescribed with antipsychotics or mood stabilizers. When medication alterations were necessary, the favoured options involved combination with antipsychotics or transitioning to alternative antidepressants. Yet, in the MDS group, following these initial modifications, the addition of mood stabilizers tended to be the more prioritized alternative.

**Conclusions:**

MDD patients with suicidal ideation or behaviour displayed distinctive demographic and clinical features. They exhibited intricate treatment patterns, a pronounced burden of illness, and an increased likelihood of relapse.

## Introduction

According to the World Health Organization (WHO), nearly 800,000 people die from suicide each year [1]. Among them, approximately 90% suffer from a mental illness, with 40–70% suffering from major depressive disorder (MDD) [2]. In China, its lifetime prevalence is 6.8% [3]. A comprehensive meta-analysis on Chinese patients with MDD revealed that the lifetime prevalence rates of suicidal ideation, suicide plans, and suicide attempts were 53.1%, 17.5%, and 23.7%, respectively [4], which were consistent with rates reported in international studies. Compared to patients without suicidal ideation (SI) or suicidal behaviour (SB), patients with MDD with SI and/or SB were at a higher risk of comorbid mental and medical conditions [5]. They consumed more antidepressants, experienced severe side effects, had poorer drug efficacy and quality of life, and required additional medical resources and expenses [5]. Meanwhile, their caregivers also bore enormous burdens, including financial and psychological [6]. Therefore, suicide in patients with MDD represents an extremely massive burden for society and their families. Early identification of suicide risk in patients with MDD and timely and efficacious intervention is an important public health issue.

Suicide in patients with MDD is a highly complex behaviour with individual differences, which can be challenging to predict and identify [7]. Studies have attempted to find associated risk factors from multiple aspects, such as demographic characteristics [8, 9] (younger age, male, smoking, unemployment, family history of mental illness and history of suicide attempts), psychosocial factors [10] (low social support), life stressors [11] (childhood and adult life adversities), comorbidities [12, 13] (borderline personality disorder and coronary heart disease), and clinical features [9, 14] (severity of depressive symptoms, presence of psychotic symptoms and melancholic depression). Although these factors are associated with a higher risk of suicide in MDD, they only provide limited predictive value. Therefore, further research aims to identify objective biological markers related to suicide in depression. Studies identified potential biological markers associated with depression-related suicide, such as interleukin-6 [15], C-reactive protein [16], the serotonin transporter gene [17], brain-derived neurotrophic factor gene [18] and abnormal DNA methylation levels [19]. However, these studies are still in the early stages and face certain challenges and limitations. The consistency and replicability of their findings require further verification. Additionally, the discovery of biomarkers necessitates larger-scale investigations.

Managing patients with MDD with suicide ideation and/or behaviour is particularly challenging owing to the difficulty in predicting and detecting suicide, urgency of the condition, need for immediate life-sustaining intervention and paucity of available treatments [20]. For high-risk patients, conventional antidepressants usually have a slow onset and often taking more than two weeks to achieve efficacy [21]. In clinical practice, some physical therapy methods, especially electroconvulsive therapy (ECT), are often used to eliminate patients’ suicidal ideation and behaviour. Studies found that ECT effectively reduced the risk of suicide [22] and might be superior to antidepressants in preventing recurrence of suicide in depressed patients [23], pending further empirical evidence. However, when considering its impact on cognition[24], some patients and their families had concerns. Ketamine was a blocker of the N-methyl-D-aspartate (NMDA, glutamate-gated ion channel) receptors. Ketamine and its isomer, es-ketamine, reduced the risk of suicide in patients in a short time, as confirmed by relevant studies [25, 26]. As the primary modalities of long-term intervention, the use of antidepressants [27], lithium [28, 29], second general antipsychotics [30] and psychotherapy (including cognitive behavioural therapy [31], dialectical behavioural therapy [32], etc.) alleviated depressive symptoms and reduce the risk of suicide death among patients. However, there is ongoing debate and controversy regarding the effectiveness of the aforementioned treatment modalities in suicide prevention for individuals with depression [32–35].

In summary, suicide is the most serious consequence of depression and requires early identification and prompt intervention. Both domestic and foreign guidelines recommend close monitoring and treatment of patients assessed for suicidal ideation and behaviour and admission to hospital for drug treatment and psychotherapy [36]. The current treatment strategies for suicidal ideation and behaviour in patients with MDD are unclear. Most randomized controlled trials (RCTs) on drugs and physical therapy for MDD excluded patients with suicidal tendencies for safety reasons [37], which made the evidence obtained not generalizable to a clinic setting. Starting from disease databases and real-world data, exploring the characteristics of individuals with SI and SB, understanding their disease burden, and seeking effective treatment strategies is crucial. This study aimed to understand and compare the characteristics and disease burden of individuals with MDD with or without suicidal ideation or behaviours in psychiatric hospitals through real-world data. Furthermore, by tracking medication adjustments during the hospitalization period, we hoped to identify the treatment pattern transitions for individuals with suicide to optimize clinical treatment pathways.

## Methods

### Data source

This descriptive, retrospective cohort study used electronic health records based on the Hospital Information System. The study investigated the clinical characteristics, treatment features and disease burden of patients with major depressive disorder with suicidal ideation or behaviour. The cohort was selected from inpatients at Beijing Anding Hospital Capital Medical University between January 2013 to December 2020. All patients were observed for a one-year follow up period after discharge to explore recurrence (rehospitalization). The inclusion criteria were: (1) patients diagnosed with major depressive disorder (by an experienced attending physician and another senior physician) according to the International Statistical Classification of Diseases and Related Health Problems, Tenth Revision (ICD-10) diagnostic criteria and (2) availability of reliable medical records, without any missing information. Exclusion criteria were: (1) those diagnosed with other mental disorders, such as schizophrenia and bipolar disorder, (2) severe physical diseases, (3) long-term hospitalized patients (>6 months), (4) patients with drug or alcohol dependence or abuse issues and (5) women who were pregnant and/or lactating.

### Patient identification

Among the included data, we screened a portion of samples that included complete discharge records with a hospitalization duration of at least > 2 weeks. Based on the presence of suicidal ideation and/or behaviour at admission (extracted from specific suicide-related fields recorded on the day of admission), the enrolled patients were divided into two groups: major depressive disorder with suicidal ideation or behaviour (MDS) and with no suicidal ideation or behaviour (MDNS). Demographic and clinical, detailed treatment and re-hospitalization information within one year were obtained from the database. Furthermore, the clinical characteristics, treatment features and disease burden were analysed between the two groups. In addition, relapse and recurrence of depression were evaluated via the one-year follow-up data after discharge.

### Treatment patterns

We included the main psychiatric medications during hospitalization, which included antidepressants (AD), antipsychotics (AP) and mood stabilizers (MS). AD was further divided into SSRI, Serotonin Norepinephrine Reuptake Inhibitor (SNRI), Norepinephrine Dopamine Reuptake Inhibitor (NDRI), Noradrenergic and Specific Serotonergic Antidepressant (NaSSA) and others based on pharmacological mechanisms. Given the severity of patients’ conditions and close monitoring in the hospital setting, there is a high probability of actively modifying medications. Opting for a relatively narrow timeframe can facilitate the identification of modifications in the therapeutic regimen, we defined the observation period as seven days to observe treatment regimens (patterns). If drug A was used continuously in combination with drug B and drugs A and B were used in combination for more than seven days, the treatment regimen was defined as drug A combined with drug B (A + B). If drugs B and A overlapped for less than seven days and the patient mainly used drug B in the next observation period (seven days), it was defined as a drug switch. Both drug combination and switch were considered as a new treatment regimen. Any change to the class of medications was reflected as a new treatment regimen, which included (1) the main medication composition changed and in-class switching was captured as a new regimen, such as an individual on escitalopram who switched to sertraline, or (2) addition of a new medication to an existing regimen, (combination therapy), such as AP and MS added to AD.

### Common data model

Data were mapped to standard concepts according to the Observational Medical Outcomes Partnership Common Data Model [38]. Furthermore, the treatment sequence analysis was performed within the Observational Health Data Sciences and Informatics framework.

### Statistical analysis

Statistical Product and Service Solutions version 26.0 (Chicago, IL, USA) was used for statistical analyses. Continuous variables did not conform to the normal distribution and homogeneity of variance. Data were presented as median and quartile ranges. A Kruskal-Wallis H-test was used to compare the differences in numerical variables between the two groups. All categorical variables were expressed as numbers and percentages (%) and compared using a chi-squared test. Two-tailed P values were used for all statistical analyses, and *p* < 0.05 was considered as statistically significant.

## Results

### Differences in demographic and clinical features between the MDNS and MDS groups

Table 1 presents the demographic and clinical characteristics and treatment burden between the MDNS and MDS groups. Regarding demographic information, no significant differences were observed between the two groups in smoking, marital status, and education level (*p* > 0.05). Compared to patients with non-suicidal ideation and/or behaviour, patients with suicidal behaviour and/or ideation (MDS) were younger (Z=-4.698, *p* < 0.001) and aged under 29 years (χ^2^ = 39.56, *p* < 0.001). In addition, the MDS group had a higher proportion of females (68.31% vs. 65.41%, χ^2^ = 7.572, *p* < 0.001) and frequency of alcohol consumption (χ^2^ = 3.995, *p* = 0.046). Regarding clinical features, the MDS group had more life stress events (42.61% vs. 38.23%, *p* < 0.001) and earlier onset age (33 years vs. 35 years, Z=-1.261, *p* = 0.207). There were no significant differences between the two groups in family history, duration of illness and psychiatric symptoms and number of hospitalizations within five years.

**Table 1:** Patients’ demographic features and clinical characteristics

Variables	MDS (*n* = 4138)	MDNS (*n* = 3848)	Z/χ2	*P value	
**Demographic features**					
Age (years)	43 (23, 57.)	45 (27, 58)	-4.698	< 0.001*	
< 18	603 (14.58)^a^	445 (11.56)^a^	39.56	< 0.001*	
18–29	800 (19.34)^a^	649 (16.87)^a^			
30–39	496 (11.99)^a^	537 (13.96)^a^			
40–49	550 (13.29)	560 (14.55)			
50–59	869 (21.01)	770 (20.01)			
≥ 60	819 (19.80)^a^	887 (23.05)^a^			
Male (%)	1311 (31.69)	1331 (34.59)	7.572	0.006*	
Alcohol consumption (yes, n, %)	544 (13.39)	439 (11.88)	3.995	0.046*	
Tobacco use (yes, n, %)	704 (17.12)	620 (16.59)	0.392	0.531	
Marital status (yes, n, %)	2427 (58.68)	2306 (60.04)	1.519	0.218	
Level of education (n, %)					
University or above	477 (36.95)	451 (37.65)	0.799	0.671	
Secondary or High school	687 (53.21)	619 (51.67)			
Elementary school or below	127 (9.84)	128 (10.68)			
**Clinical characteristics**					
Stress events (yes, n, %)	1763 (42.61)	1471 (38.23)	15.856	< 0.001*	
Family history (yes, n, %)	543 (13.12)	508 (13.20)	0.011	0.916	
Duration of illness (years)	3.00 (0.83, 10.00)	3.00 (0.92, 10.00)	-1.261	0.207	
Age of onset (years)	33 (18, 49)	35 (20, 51)	-5.218	< 0.001*	
Psychotic symptoms (yes, n.%)	768 (18.56)	766 (19.91)	2.330	0.127	
Number of hospitalizations within five years prior to admission (n, %)					
1 time	3373 (81.51)	3201 (83.19)	5.76	0.218	
2 times	591 (14.28)	486 (12.63)			
3 times	123 (2.97)	118 (3.07)			
4 times	35 (0.85)	33 (0.86)			
≥ 5 times	16 (0.39)	10 (0.26)			
**Treatment Burden**					
ECT (yes, n, %)	2347 (56.72)	1682 (43.71)	134.946	< 0.001*	
Hospital stays (days)	28.00 (20.00, 37.00)	28.00 (19.00, 37.00)	-1.778	0.075	
Re-hospitalization within 6 months (yes, n, %)	470 (11.36)	343 (8.91)	13.029	< 0.001*	
Re-hospitalization within 7 to 12 months (yes, n, %)	244 (5.90)	195 (5.07)	2.638	0.104	

Note: Continuous data are presented as median (lower quartile, upper quartile) and compared via Kruskal–Wallis H test. Categorical data are indicated by (number/total number) and percentage (%) and compared via chi-squared test

^*^P-value was statistically significant

^a^ Significant differences between the two groups after post-hoc analysis

Abbreviations: MDS, major depressive disorder with suicidal ideation or behaviour; MDNS, major depressive disorder with none suicidal ideation or behaviour; ECT, electroconvulsive therapy

### Treatment burden between the MDNS and MDS groups

Regarding treatment burden (Table 1), a higher proportion of patients in the MDS group were combined with ECT treatment (56.72% vs. 43.71% *p* < 0.001), which suggested that treatment in the MDNS group was more challenging. In addition, the proportion of rehospitalization within six months after discharge was also significantly higher in the MDS group than that in the MDNS group (11.36 vs. 8.91%, *p* < 0.001). Furthermore, rehospitalization rate between the two groups in the long term (7–12 months) showed no difference.

### Treatment pattern between the MDNS and MDS groups

Antidepressants were the main treatment of depression. Hence, we considered antidepressants as the core of treatment classification. The pharmacal treatments were grouped into six categories, namely AD, AD + AD, AD + AP, AD + MS, AD + MS + AP and others. When antidepressants use was further examined, we categorized antidepressants into SSRI, SNRI, NaSSA, NDRI and others.

#### Treatment patterns of medication class in different regimens

In both the MDS and MDNS groups, the majority of patients (61.82% vs. 62.35%) showed improvement and were discharged after one medication regimen. A small percentage (< 6%) received four or more than five regimens. Approximately 32% of patients in the MDS and MDNS groups received two or three regimens (Table 2). There was no statistically significant difference in the number of treatment rounds between the two groups (χ^2^ = 1.720, *p* = 0.787), as shown in Table 2. Table 3 presents the sequence of the treatment classes. In both the MDS and MDNS groups, the most common treatment was AD + AP (approximately 40%), followed by monotherapy of AD (15–36%), AD + AD (8–17%) and others (6–14%). The least common regimens were AD + MS + AP (2–6%) and AD + MS (2–10%). In the MDS group, the proportion of AD monotherapy gradually decreased as treatment progressed (from the first to fifth regimen). The treatment of AD + AD increased in the second and third regimens, subsequently decreased in the fourth regimen and increased again in the fifth regimen. A similar trend was also observed in the MDNS group. The combination of AD + AP was used most frequently in both groups. The proportion of AD + MS treatment class fluctuated within the regimens. In the MDS group, the AD + MS + AP treatment class increased with an increase in the number of regimens. However, this change did not present in the MDNS group. Use of ECT procedures was more (>50%) in MDS group compared with MDNS group (less than 40%).

**Table 2:** Comparison of treatment regimens between the MDS and MDNS groups

	MDS	MDNS	χ2	*P value
Regimens during hospitalization	n, %	n, %		
1st regimen	2508 (61.82)	2332 (62.35)	1.720	0.787
2nd regimen	848 (20.90)	765 (20.45)		
3rd regimen	489 (12.05)	429 (11.47)		
4th regimen	152 (3.75)	154 (4.12)		
5th regimen	60 (1.48)	60 (1.60)		

Abbreviations: MDS, major depressive disorder with suicidal ideation or behaviour; MDNS, major depressive disorder with none suicidal ideation or behaviour

**Table 3:** Proportion of patients receiving treatment within each regimen

	MDS					MDNS				
Treatment	1st	2nd	3rd	4th	≥ 5th	1st	2nd	3rd	4th	≥ 5th
**Pharmacotherapy** (n,%)										
AD	915 (36.48)	270 (31.84)	115 (23.52)	42 (27.63)	13 (21.67)	796 (34.13)	229 (29.93)	91 (21.21)	39 (25.32)	9 (15.00)
AD + AD	203 (8.09)	121 (14.27)	84 (17.18)	19 (12.50)	9 (15.00)	236 (10.12)	104 (13.59)	59 (13.75)	18 (11.69)	10 (16.67)
AD + AP	1021 (40.71)	307 (36.20)	203 (41.51)	66 (43.42)	24 (40.00)	985 (42.24)	295 (38.56)	205 (47.79)	66 (42.86)	28 (46.67)
AD + MS	86 (3.43)	25 (2.95)	22 (4.50)	3 (1.97)	6 (10.00)	58 (2.49)	13 (1.70)	10 (2.33)	3 (1.95)	4 (6.67)
AD + MS + AP	123 (4.90)	50 (5.90)	25 (5.11)	12 (7.89)	4 (6.67)	88 (3.77)	20 (2.61)	18 (4.20)	7 (4.55)	1 (1.67)
Others	160 (6.38)	75 (8.84)	40 (8.18)	10 (6.58)	4 (6.67)	169 (7.25)	104 (13.59)	46 (10.72)	21 (13.64)	8 (13.33)
**Physiotherapy** (n, %)										
ECT	2180 (52.90)	2133 (51.76)	1295 (31.42)	533 (12.93)	252 (6.12)	1523 (39.89)	1472 (38.55)	906 (23.73)	406 (10.63)	190 (4.98)

Abbreviations: MDS, major depressive disorder with suicidal ideation or behaviour; MDNS, major depressive disorder with none suicidal ideation or behaviour; AD, antidepressants; AP, antipsychotics;MS, mood stabilizers;ECT, electroconvulsive therapy

#### **Treatment patterns of antidepressants in the different regimens**

After the psychiatric medication class was observed between the MDS and MDNS groups, we further explored the pathways of antidepressant use. Antidepressants were analysed by subclass (SSRI, SNRI, NaSSA, NDRI and others). We defined that as long as the composition of antidepressants changed during the observation, it was regarded as a new regimen. Changing from escitalopram to sertraline was counted as a new regimen. Only when AP and MS were newly added, they could be considered as a new regimen. Furthermore, if there was a change in composition within a medication class, it did not count as switch. If the medication changed from escitalopram combined with quetiapine (SSRI + AP) to escitalopram combined with olanzapine, it was not considered a new regimen as the types of antidepressants had not changed. Hence, the overall treatment regimen was still SSRI + AP. Escitalopram initially followed by quetiapine was considered a change in treatment regimen as this was from SSRI to SSRI + AP.

Detailed treatment pathway of antidepressants in both groups are displayed in Tables 4 and 5, respectively. In patients with no switch (switch time = 0), the top five treatment classes in MDS group were: SSRI + AP, SSRI, SNRI + AP, SNRI and SNRI + AP + MS. Furthermore, AP alone ranked higher in the MDNS group. In patients who experienced one switch in both groups, the most common switching scheme was combination therapy, such as addition of an AP or MS or NaSSA to an existing SSRI or SNRI regimen. For patients with SSRI/SNRI + AP, switching to another antidepressant or a combination with MS was more used in sequencing therapy. For patients on AP alone, antidepressants (SSRI or SNRI) or MS were added in the next step of treatment. Among patients who underwent two switches, the main treatment strategy was still the conversion of the antidepressant classes or addition of AP and MS or NaSSA to the existing SSRI or SNRI. In addition, some patients no longer used antidepressants and instead used MS + AP. The switching strategies were similar between the MDS and MDNS groups.

**Table 4:** Medication pathway of antidepressants for the MDS group in each regimen

Switch	Top5	1st regimen					
		Medication	n(%)				
0 time (*n* = 2661)	1	SSRI + AP	730 (27.43)				
	2	SSRI	643 (24.16)				
	3	SNRI + AP	273 (10.26)				
	4	SNRI	178 (6.69)	2nd regimen			
	5	SSRI + AP + MS	106 (3.98)	Medication	n(%)		
1 time (*n* = 850)	1	SSRI	177 (20.82)	SSRI + AP	111 (62.71)		
				SSRI + MS	23 (12.99)		
				SSRI + NaSSA	19 (10.73)		
	2	SSRI + AP	128 (15.06)	SSRI + AP + MS	44 (34.38)		
				SSRI	22 (17.19)		
				AP	15 (11.72)		
	3	AP	66 (7.76)	SSRI + AP	27 (40.91)		
				AP + MS	24 (36.36)		
				SNRI + AP	9 (13.64)		
	4	SNRI	58 (6.82)	SNRI + AP	37 (63.79)		
				NaSSA + SNRI	8 (13.79)		
				SNRI + MS	3 (5.17)		
	5	SNRI + AP	45 (5.29)	SNRI	10 (22.22)		
				NaSSA + SNRI + AP	9 (20.00)	3rd regimen	
				SNRI + other + AP	7 (15.56)	Medication	n(%)
2 times (*n* = 405)	1	SSRI + AP	86 (21.23)	SSRI + SNRI + AP	21 (24.42)	SNRI + AP	20 (95.24)
				SSRI + AP + MS	20 (23.26)	AP + MS	13 (65.00)
				SSRI + SSRI + AP	16 (18.60)	SSRI + AP	15 (93.75)
	2	SSRI	71 (17.53)	SSRI + SNRI	17 (23.94)	SNRI	15 (88.24)
				SSRI + AP	14 (19.72)	SSRI + AP + MS	6 (42.86)
						SNRI + AP	3 (21.43)
				SSRI + SSRI	11 (15.49)	SSRI	9 (81.82)
	3	SSRI + NaSSA	30 (7.41)	SSRI + NaSSA + AP	7 (23.33)	SSRI + AP	3 (42.86)
				SSRI + NaSSA + SNRI	6 (20.00)	NaSSA + SNRI	4 (66.67)
	4	SNRI + AP	26 (6.42)	SSRI + SNRI + AP	8 (30.77)	SSRI + AP	6 (75.00)
				SNRI + AP + MS	6 (23.08)	AP + MS	4 (66.67)
	5	SNRI	22 (5.43)	SNRI + AP	7 (31.82)	SSRI + SNRI + AP	1 (14.29)
						SSRI + AP + MS	1 (14.29)
				SSRI + SNRI	5 (22.73)	SSRI	3 (60.00)
	5	NaSSA	22 (5.43)	SSRI + NaSSA	9 (40.91)	SSRI	7 (77.78)
				NaSSA + SNRI	6 (27.27)	SNRI	3 (50.00)

Abbreviations: AD, antidepressants; AP, antipsychotics;MS, mood stabilizers; SSRI, Selective Serotonin Reuptake Inhibitors; SNRI, Serotonin-Norepinephrine Reuptake Inhibitor; NDRI, Norepinephrine Dopamine Reuptake Inhibitor

**Table 5:** Medication pathway of antidepressants for the MDNS group in each regimen

Switch	Top5	1st regimen					
		Medication	n(%)				
0 time *n* = 2510	1	SSRI + AP	781 (31.12)				
	2	SSRI	516 (20.56)				
	3	SNRI + AP	219 (8.73)				
	4	SNRI	158 (6.29)	2nd regimen			
	5	AP	129 (5.14)	Medication	n(%)		
1 time (*n* = 762)	1	SSRI	141 (18.50)	SSRI + AP	98 (69.50)		
				SSRI + NaSSA	17 (12.06)		
				SSRI + others	8 (5.67)		
	2	SSRI + AP	119 (15.62)	SSRI	36 (30.25)		
				SSRI + AP + MS	29 24.37)		
				AP	17 (14.29)		
	3	AP	92 (12.07)	SSRI + AP	69 (75.00)		
				AP + MS	15 (16.30)		
				SNRI + AP	4 (4.35)		
	4	SNRI	50 (6.56)	SNRI + AP	33 (66.00)		
				NaSSA + SNRI	4 (8.00)		
	5	SNRI + AP	41 (5.38)	SNRI	13 (31.71)		
				SNRI + AP + MS	9 (21.95)	3rd regimen	
				NaSSA + SNRI + AP	7 (17.07)	Medication	n(%)
2 times (*n* = 335)	1	SSRI + AP	65 (19.40)	SSRI + SNRI + AP	21 (32.31)	SNRI + AP	20 (95.24)
				SSRI + AP + MS	17 (26.15)	AP + MS	11 (64.71)
				SSRI + SSRI + AP	8 (12.31)	SSRI + AP	7 (87.50)
	2	SSRI	56 (16.72)	SSRI + SNRI	14 (25.00)	SNRI	12 (85.71)
				SSRI + AP	13 (23.21)	SSRI + AP + MS	4 (30.77)
						SSRI/AP	4 (30.77)
	3	SNRI + AP	25 (7.46)	SSRI + SNRI + AP	7 (28.00)	SSRI + AP	7 (100.00)
				SNRI + AP + MS	5 (20.00)	AP + MS	2 (40.00)
				NaSSA + SNRI + AP	4 (16.00)	SNRI + AP	3 (75.00)
	4	SSRI + NaSSA	24 (7.16)	SSRI + NaSSA + AP	9 (37.50)	SSRI + AP	8 (88.89)
				SSRI + SSRI + NaSSA	5 (20.83)	SSRI + NaSSA	4 (80.00)
				SSRI + NaSSA + SNRI	5 (20.83)	NaSSA + SNRI	4 (80.00)
	5	AP	20 (5.97)	SSRI + AP	13 (65.00)	SSRI + AP + MS	4 (30.77)
						SSRI	3 (23.08)

Abbreviations: AD, antidepressants; AP, antipsychotics;MS, mood stabilizers; SSRI, Selective Serotonin Reuptake Inhibitors; SNRI, Serotonin-Norepinephrine Reuptake Inhibitor; NDRI, Norepinephrine Dopamine Reuptake Inhibitor. NaSSA, Noradrenergic and Specific Serotonergic Antidepressant

When the classes of antidepressants used at discharge and admission were compared, antidepressant regimens were different (*p* < 0.05) among the group, as shown in Table 6. In the MDS group, compared with admission, the proportion of SSRI and decreased (from 53.57 to 49.92%) and proportion of SNRI increased (from 19.89 to 22.06%) at discharge. In addition, the proportion of not using AD increased slightly (from 7.18 to 9.45%). In the MDNS group, the proportion of SNRI use increased (19.15–21.25%), proportion of SSRI use did not change and proportion of patients who did not take AD decreased (9.75–8.20%). Compared with the MDNS group, the SNRI regimen were more received and a higher percentage of patients did not use AD at discharge. The MDS group was more likely to choose the SNRI regimen and prone to not use antidepressants at discharge.

**Table 6:** Comparison of antidepressant distribution at admission and discharge

Antidepressant Class	MDS		χ2	P value	MDNS		χ2	P value
	Admission	Discharge			Admission	Discharge		
SSRI (n, %)	2580 (53.57)^a^	2371 (49.92)^a^	28.795	< 0.001*	2269 (50.75)	2268 (50.94)	12.06	0.034*
SNRI (n, %)	958 (19.89)^a^	1048 (22.06)^a^			856 (19.15)^a^	946 (21.25)^a^		
NaSSA (n, %)	669 (13.89)	615 (12.95)			671 (15.01)	641 (14.40)		
NDRI (n, %)	91 (1.89)	83 (1.75)			71 (1.59)	62 (1.39)		
Others (n, %)	172 (3.57)	184 (3.87)			168 (3.76)	170 (3.82)		
Without AD (n, %)	346 (7.18)^a^	449 (9.45)^a^			436 (9.75)^a^	365 (8.20)^a^		

Abbreviations: SSRI, Selective Serotonin Reuptake Inhibitors; SNRI, Serotonin-Norepinephrine Reuptake Inhibitor; NDRI, Norepinephrine Dopamine Reuptake Inhibitor. NaSSA, Noradrenergic and Specific Serotonergic Antidepressant

## Discussion

Suicide is the most serious adverse outcome for patients with MDD. It requires early identification and timely intervention. However, the treatment of patients with depression who experience suicidal ideation and behaviour remains highly challenging. This study used real-world data to understand the treatment and clinical characteristics as well as disease burden in patients who were depressed and suicidal ideation and behaviour. Patients with major depressive disorder with suicidal ideation and behaviour had unique demographic and clinical characteristics. They required more frequent physical treatments and had a higher relapse rate within six months. Regarding treatment, approximately 20% required three or more treatment courses. Furthermore, over 90% used antidepressant medications alone or in combination, and SSRIs were most commonly used. Regardless of the initial treatment phase or throughout the entire hospitalization period, the combination of antidepressants with antipsychotics or mood stabilizers was frequently prescribed. ECT was considered an effective treatment for suicide treatment. Our study found a higher proportion of patients with suicidal tendencies underwent ECT during treatment period. Regarding treatment pathways, switching to antipsychotics or mood stabilizers in combination with antidepressants was the preferred option, followed by changing the type of antidepressant medication.

Suicide, a complex behaviour, was influenced by multiple interacting factors [7]. Studies have not comprehensively examined various characteristics among patients, which could result in inconsistent findings [9]. We found that younger age (≤ 29 years older), being female, and a history of alcohol consumption were associated with a higher likelihood of suicidal ideation or behaviour among patients with depression. According to data from the WHO, suicide was the second leading cause of death among those aged 15–29 years. These people often faced high levels of social stress, experienced emotional volatility, and had limited understanding and coping skills when it came to depression [39]. Due to a lack of consistent definitions, the correlation between gender and suicide related to depression remains unclear. Studies also found that male individuals with depression had a higher risk of suicide [9] in the general population. Furthermore, females tended to exhibit suicidal ideation and suicide attempts, whereas males were more likely to die by suicide [7].

The stress-diathesis model of suicide suggests that childhood abuse experiences and stressful events in adulthood serve as initial and precipitating factors for suicide respectively. Additionally, both are risk factors for the occurrence of depression and subsequent suicidal behaviour [40]. Increased risk of suicide associated with stress may be related to dysregulation of the hypothalamic–pituitary–adrenal axis [41], elevated inflammatory levels [42], enhanced glutamate system function [43] and other factors [44]. We analysed the precipitating factors of patients’ current onset and found that patients with depression who experienced stressful life events were more likely to have suicidal ideation or behaviour. Although we did not separate the timing of stress occurrence, tracking patients’ stress history through medical records and analysing laboratory indicators, especially those related to stress, such as inflammatory factors and hormones, would provide additional evidence for identifying depression-related suicide.

Due to the presence of suicide, the treatment for these patients requires more urgent and proactive intervention measures. ECT is a first-line treatment for rapidly relieving suicidal ideation and behaviour in hospitalized patients with MDD [45]. Multiple studies reported significant improvements in reduction of suicidal ideation, tendencies and behaviour in patients with depression who received ECT [46, 47]. We also found a higher proportion of patients with suicidal ideation or behaviour received ECT treatment compared to the positive control group with depression. Further analysis revealed that these patients initiated ECT treatment earlier and received it for a longer duration. This suggested that the treatment of depression with suicide was more challenging. Since the cost of ECT treatment was higher compared to medication, these patients bore a heavier disease burden. However, the effectiveness of ECT was not sustained. Some studies suggested an increased risk of suicide after ECT treatment [48]. Hence, managing patients with suicide risk may require longer and further persistent treatment strategies. Therefore, providing appropriate and effective treatment to these patients is beneficial for improving their mental health condition and reducing risk of suicide.

We observed changes in medication prescription strategies across different regimens. The proportion of patients who used antidepressant monotherapy was low in both groups. More patients received a combination of antipsychotics or mood stabilizers, especially with the AD + AP treatment program, which showed a higher utilization rate across each regimen. The combination of various treatment plans could reflect patients’ clinical characteristics (comorbid psychotic symptoms or paranoid personality), comorbid conditions (anxiety or sleep disorders) and severity and refractoriness of suicidal ideation or behaviour [20]. Further analysis that combined medication dosage or blood drug concentration with clinical symptoms could provide a better explanation for the rationality of different combination strategies.

In subsequent analyses, we attempted to identify a subgroup of patients who required multiple medication changes and tracked their medication switching patterns to observe changes in the treatment pathway. When the treatment outcomes were unsatisfactory after one round, most patients preferred combination with antipsychotics. This could be attributed to their clinical characteristics and comorbid conditions and also the synergistic effect of combining antipsychotics [49, 50]. Additionally, for the MDS group, the proportion of combining mood stabilizers over the existing treatment plan was higher than that in the MDNS group. This may be due to the relatively clear efficacy of mood stabilizers (especially lithium) in preventing suicide [28]. Nevertheless, the proportion of patients who combined mood stabilizers was much lower than those who combined antipsychotics, possibly due to concerns among clinicians regarding the risks of lithium overdose and other safety events [51]. Furthermore, increasing evidence has suggested that antipsychotics (chlorpromazine, quetiapine, etc.) could reduce the risk of suicide in patients with depression [30].

Most current treatment guidelines suggest that SSRIs should be the first choice and, in the case of non-response, an antidepressant from a different pharmacological class should be used. There are relatively few data to inform the choice of which antidepressant from which class should be used in non-responders. Some studies have suggested that SNRIs should be the second step choice [52]. Upon discharge, it is conceivable that patients may not have been commenced on antidepressants, a decision potentially underpinned by considerations of an associated escalation in suicide risk attributable to the utilization of such pharmacological interventions. This is particularly salient in individuals with a predisposition to high impulsivity, where the prescription of antidepressants necessitates an augmented level of clinical vigilance. Additionally, given the observational nature of this real-world study, it cannot be discounted that a subset of patients may have been discharged prior to the initiation of antidepressant therapy.

In the one-year follow-up after discharge, we found that within the first six months after discharge, patients with depression who had suicidal thoughts or behaviours were more likely to experience a relapse. Recent suicide attempts were identified as a high-risk factor for recurrent suicidal ideation or behaviour in patients with depression [53]. Research demonstrated that approximately 14% of patients had another suicide attempt within six months after the previous attempt [54], which was similar to the findings of other longitudinal studies that lasted one year or longer [55]. The logistic regression analysis identified only one variable predicting a potential recurrence of suicide, which was receiving relevant psychological or medication treatment at admission [56]. Additionally, patients who were hospitalized in a psychiatric specialty hospital were more likely to attempt suicide again after discharge [56], which suggested a critical period for suicide intervention following discharge.

Our findings have implications for clinical practice. MDD patients with suicidal ideation or behavior display distinct clinical features, such as an earlier age of onset and often presenting with stress events, indicating that they may experience heightened psychological stress and social challenges. MDS patients require more intricate medication regimens, incur higher hospitalization costs, and exhibit higher relapse rates, underscoring the necessity for more comprehensive and long-term treatment approaches. When adjusting treatment plans, the addition of antipsychotic medications or mood stabilizers appears to be the preferred course of action, followed by changing the type of antidepressant prescribed. In essence, this study offers a detailed insight into the clinical profile and treatment requirements of individuals with MDD and suicidal ideation or behavior, providing valuable guidance for enhancing diagnostic and treatment pathways, implementing early interventions, and developing effective treatment strategies in the future.

This study has some limitations. First, due to the early stage of construction and operation of the large database platform at Beijing Anding Hospital, Capital Medical University, data collection was not comprehensive enough to include indicators related to disease severity, previous suicide attempts and cognitive function, which were relevant to the risk of suicide in patients with depression. Further validation can be conducted based on these findings. Second, this study was a single-centre study, which may not have covered all patients’ medical and treatment data, which could have potentially led to research bias when exploring relapse and recurrence. Third, this study did not include the treatment plans used by patients in outpatient and other healthcare institutions after discharge. Therefore, we cannot determine the long-term effectiveness of the treatment plan at discharge, as previous studies reported that nearly 50% of patients with depression with suicidal ideation or behaviour received at least three different treatment plans within one year [20].

## Conclusion

Patients with depression accompanied by suicidal ideation or behaviour have unique demographic and clinical characteristics. Their treatment plans are more complex, burden of illness is heavier, and likelihood of disease relapse is higher.

## Data Availability

The data used in this study are available from the corresponding author upon reasonable request.
